# Robotic end effector for decompression of tension pneumothorax

**DOI:** 10.1007/s11548-026-03710-3

**Published:** 2026-06-02

**Authors:** Carolin Müller, Robert Roth, Lars Wagner, Christoph Parhofer, Peter Biberthaler

**Affiliations:** 1 TUM School of Medicine and Health, University Hospital rechts der Isar, Department of Trauma Surgery, Technical University of Munich, Munich, Germany; 2 TUM School of Medicine and Health, University Hospital rechts der Isar, Department of Surgery, Technical University of Munich, Munich, Germany; 3 TUM School of Engineering and Design, Institute of Micro Technology and Medical Device Technology, Technical University of Munich, Munich, Germany

**Keywords:** Tension pneumothorax, End effector, Robotics, Needle–catheter motion

## Abstract

**Background:**

This work aims to develop and evaluate a robotic end effector capable of performing needle decompression for tension pneumothorax, enabling life-saving intervention in the absence of on-site medical personnel.

**Methods:**

A compact modular system was designed featuring a single-actuator control for relative needle–catheter motion, automated sterile pickup, positioning via ultrasound and a quick-switch interface for tool exchange. Experimental validation focused on mechanical performance through critical load, friction, and needle-handling tests using ex vivo porcine thoracic tissue with a simulated pleural pressure model.

**Results:**

The end effector, including its needle-actuating gripper, was capable of delivering the estimated 30 N insertion force required for thoracic decompression. The gripper design further demonstrated structural robustness, withstanding peak axial loads exceeding 290 N. In realistic needle-handling experiments, 10 out of 11 valid attempts (90.9%) successfully achieved complete insertion, simulated decompression, and correct needle retraction, with the catheter remaining in place throughout the procedure.

**Conclusion:**

This paper proposes the first end effector design capable of performing robotic needle decompression for tension pneumothorax, enabling emergency intervention without on-site medical personnel. The proposed design demonstrates reproducible performance and procedural feasibility for robotic tension pneumothorax decompression.

## Background

Tension pneumothorax (tPTX) is a life-threatening condition caused by air trapped under pressure in the pleural cavity, most commonly due to traumatic chest injuries such as motor vehicle accidents or penetrating wounds [1]. A one-way valve effect leads to progressive pressure buildup, lung collapse, impaired venous return, reduced cardiac output, and ultimately circulatory failure [2]. The development of a tension pneumothorax can be fatal within minutes [3]. The preliminary diagnosis of a tPX is established by a physician on the basis of a characteristic injury pattern, clinical deterioration of the patient, and device-based confirmation of a pneumothorax. Needle decompression can temporarily relieve the pressure and extend survival until definitive hospital treatment is available. Needle decompression is performed by inserting a needle–catheter system into the intercostal space, followed by removal of the needle. The catheter remains in situ to relieve pressure and prevent recurrence of a tension component. The puncture site is identified using anatomical landmarks and palpation. Successful decompression is generally confirmed by clinical improvement or by the release of air. A retrospective study of 264 trauma deaths in Berlin identified isolated tPTX as the cause of death in 4 of 14 cases deemed survivable with appropriate care [4], underscoring the critical importance of timely decompression for survival.

In situations where medical personnel are scarce, such as in rural or military environments, the ability to rescue injured patients without relying on on-site personnel is becoming increasingly important. Recent work suggests that rapid aerial transport of patients to the hospital via drones equipped with diagnostic and treatment capabilities could improve prehospital survival of trauma patients[5]. As trained medical personnel is unavailable in these unmanned systems, immediate telerobotic in-air intervention for time-critical procedures such as tPTX treatment is essential during transport to ensure patient survival [6].

For such telerobotic-assisted first aid procedures, a few specialized robotic end effectors have been developed, for example, tools for safe robotic cutting of clothing [7], as well as tools for injection, oxygenation, and hemostasis [8]. However, targeting robotic needle decompression for tPTX is limited to preliminary investigation of insertion forces [9]. Extensive research exists to develop robotic systems for related tasks such as for blood sampling, placement of peripheral intravenous (IV) catheters, needle biopsy, and many others. Unlike automated venepuncture, where only a single needle is inserted and withdrawn, tPTX decompression involves a more complex sequence. After the initial puncture, the needle must be retracted while the catheter remains in place to allow continuous decompression of the pleural space. This type of two-part actuation, the separate control of the needle and catheter, is also necessary in systems for IV catheter placement. For example, HaemoBot [10] was designed to perform full robotic IV catheterization, and VeniBot [11] achieved a similar function using separate linear drives for the needle and catheter. However, in contrast with IV catheterization, tPTX decompression requires longer needles and deeper insertion paths, which impose additional demands on the actuation system. To accommodate these requirements, robotic mechanisms designed for longer needle insertions have been investigated in other medical applications. Lin et al. [12] developed a brachytherapy system capable of inserting a long stylet–catheter assembly. In this system, the relative movement between the stylet and catheter is achieved using two independent linear actuators. Similarly, Chen et al. [13] presented a robotic device employing a rack-and-pinion drive to insert an 80*mm* needle, similar in length to those used for tPTX decompression, for use in biopsies and central venous catheterization. However, this system was designed for single-component needle insertion and did not include a catheter-handling mechanism, making it unsuitable for tPTX decompression procedures that require independent motion of both components.

**Fig. 1:**
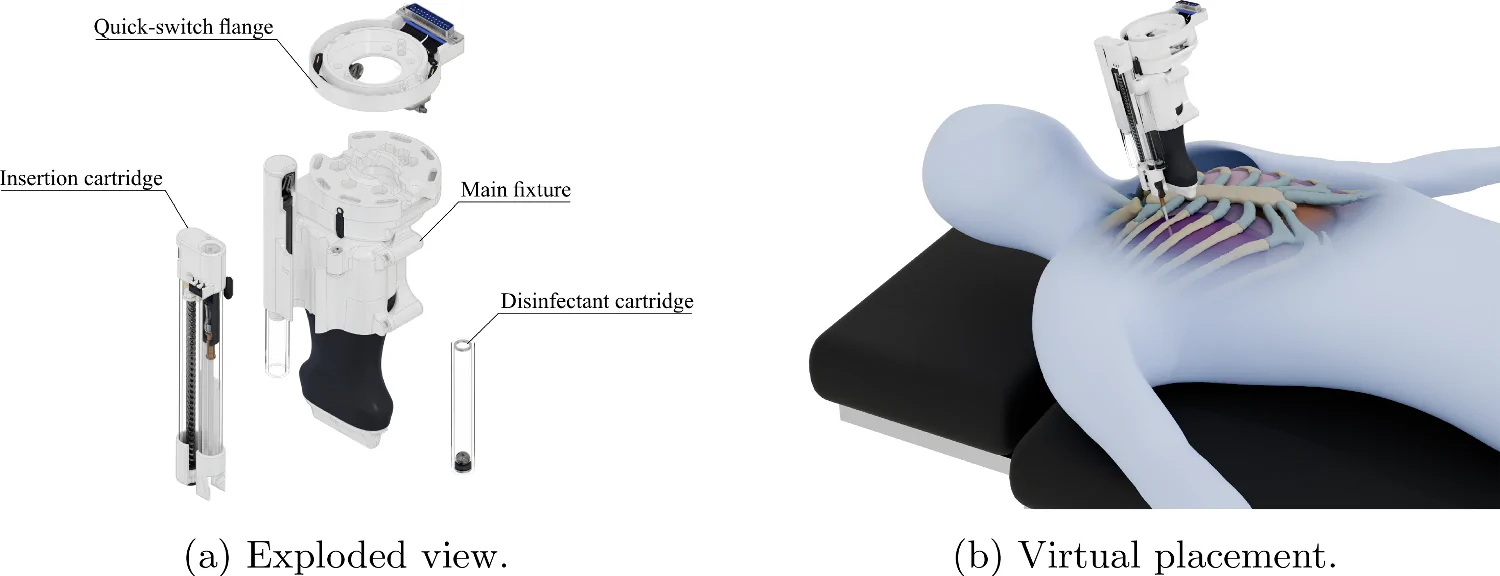
Robotic end effector for tPTX decompression. (**a**) The end effector consists of four subassemblies: an insertion cartridge, a quick-switch flange, a disinfectant cartridge, and a main fixture to which an ultrasound probe is attached. (**b**) Virtual placement of the end effector for needle decompression at the second intercostal space

To conclude, tPTX remains a critical contributor to preventable trauma deaths. Robotic systems offer a promising solution to enable timely intervention during transport without the presence of medical personnel. However, current robotic approaches lack end effectors capable of handling both the complex needle–catheter sequence required for tPTX decompression and the integration of diagnostic imaging like ultrasound for accurate localization of the puncture site. Existing research regarding needle insertion has largely focused on simpler, one-part insertions that often require manual assistance and pre-modified tools. Therefore, there is a clear need to investigate and develop a robotic end effector that can perform needle decompression, thereby enabling life-saving intervention in remote or resource-limited environments. The main contributions of this paper are:

- We present the first end effector design capable of performing robotic needle decompression for tension pneumothorax guided by ultrasound, enabling emergency intervention without on-site medical personnel (see Fig. 1).
- We introduce a compact, modular system featuring single-actuator control for relative needle–catheter motion, automated sterile pickup directly from packaging, and a quick-switch interface for tool exchange.
- We demonstrate successful robotic needle–catheter insertion, decompression, and retraction in a simulated thoracic environment using biological tissue.

## Methods

### Operational considerations

Several guidelines (Advanced Trauma Life Support (ATLS), International Trauma Life Support (ITLS)) recommend immediate decompression of suspected tPTX, preferably by needle decompression due to its simplicity. The Tactical Combat Casualty Care (TCCC) guideline [14] specifies insertion at the 2nd–3rd intercostal space mid-clavicular or 4th–5th anterior axillary, at the superior edge of the rib. Clinical studies have reported that needle decompression at the 2nd–3rd intercostal space, mid-clavicular line, can fail in up to 38% of cases [15]. In response, current trauma management guidelines such as ATLS recommend performing needle decompression at the 4th or 5th intercostal space along the anterior axillary line, which has been shown to reduce failure rates to approximately 10–15% [16].

Nevertheless, in the context of this work, the 4th–5th intercostal space at the anterior axillary line is impractical, as this region is typically obstructed by the patient’s arms during transport. Consequently, anterior access to the 2nd–3rd intercostal space at the mid-clavicular line was selected as the preferred site for decompression in this study.

The selection of optimal equipment for needle decompression remains a subject of debate. The following sections therefore examine suitable equipment for robotic localization and decompression, outline the intended robotic workflow for performing the decompression procedure, and address the challenges associated with coordinating the relative motion between the needle and catheter before presenting the mechanical design of the proposed system.

#### Optimal equipment for needle decompression

Equipment used for tPTX decompression includes standard IV catheters, specialized needle–catheter units, and Veress-type needles. Leatherman et al. [17] observed that 10-gauge catheters are more effective and reliable than 14-gauge catheters. The length of the needles is also controversial, as shorter devices carry a risk of failure and longer ones carry a risk of injury. A recent meta-analysis by Ahmad et al. [1] recommends needles with a length of 7 cm. Based on these findings, the robotic setup in this study employs a commercially available chest decompression needle with 10 gauge and 8.25 cm length (TPAK chest decompression needle, TyTek Medical), which offers sufficient insertion depth to accommodate variations in chest wall thickness.

Beyond needle type considerations, reliable identification of affected side of the thorax is critical. A tension pneumothorax originates from a pneumothorax that develops a tension component. Therefore, localization of the affected side requires identification of the pneumothorax side. A pneumothorax can be identified through several diagnostic approaches depending on the clinical context. In trauma care, ultrasound has emerged as a particularly valuable tool. The extended Focused Assessment with Sonography for Trauma (eFAST) is now widely established as the standard examination protocol for detecting critical thoracic injuries, where the absence of pleural sliding during respiration serves as a key indicator of pneumothorax [3]. Furthermore, the feasibility of performing (eFAST) under in-flight conditions with real-time telementoring has been demonstrated [18], confirming its reliability even in operationally challenging environments. For these reasons, the proposed robotic setup incorporates an ultrasound probe to enable pneumothorax imaging. A pneumothorax is visualized by ultrasound imaging of an intercostal space. This image can also be used to identify the optimal puncture site for needle decompression at the superior edge of the rib. Ultrasound is therefore additionally used to precisely localize the puncture site.

#### Robotic workflow

The intended robotic workflow envisions control of the end effector by a remotely located physician via teleoperation, who maintains ultimate decision-making responsibility. An additional 3D camera mounted above the patient within the transport vehicle provides information on the patient’s position. To perform needle decompression, the robot first installs the decompression tool, replacing any previously used tool (e.g., for clothing removal). The robot opens the sterile packaging and picks up a needle–catheter unit. Using 3D camera input, the teleoperating physician selects a rough insertion site. The 3D camera ensures a perpendicular approach to the skin surface. Ultrasound coupling between the probe and the skin is achieved with disinfection fluid, after which the teleoperating physician fine-tunes the insertion site. The ultrasound probe is positioned perpendicular to the rib axis so that the intercostal space and two adjacent ribs are visible, ensuring insertion at the superior rib edge.

**Fig. 2:**
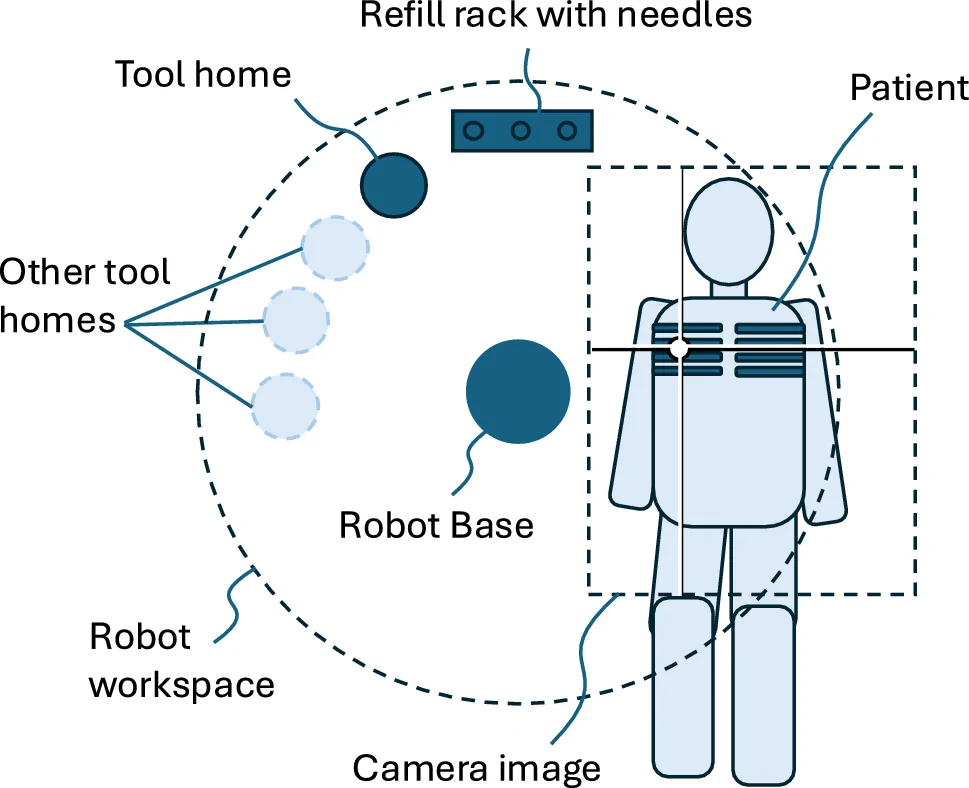
Proposed robotic setup. Top view of conceptual environment around patient. Different end effector tools can be picked up from respective tool homes. Needle–catheter units are picked up at the refill rack. 3D camera shows patient position

Once the insertion point is confirmed by the teleoperating physician, the robot inserts the needle and catheter along the planned axis. Following TCCC guidelines, the needle remains in place for 5–10 s after pleural puncture before retraction, leaving the catheter in situ. Decompression success is verified by the improvement in vital signs. The robot discards the needle while the teleoperating physician monitors the patient. If the first attempt fails, the robot loads a new needle–catheter unit, and the process repeats. Figure 2 shows a conceptual illustration of the proposed robotic setup on the patient side.

#### Task decomposition

The independent handling of needle and catheter is the central challenge in replicating the clinical decompression procedure with a robotic end effector. In our approach, a single linear actuator with a needle and catheter gripper is used to drive both the needle and the catheter during insertion, thereby reducing system complexity. Since the needle and catheter move together during the insertion phase, the catheter only needs to be separated at the final insertion depth. For this purpose, the catheter gripper is positioned at a fixed location close to the patient. During insertion, the actuator moves only the needle gripper, while the catheter remains guided by the fixed gripper. After the pleural space is reached, the needle is retracted in a controlled manner, while the catheter is held securely in place by the fixed gripper at the bottom of the end effector. This catheter gripper can be implemented either as an active gripping mechanism or as a passive guiding element.

#### Insertion force and velocity constraints

To establish force requirements, we conducted experimental measurements of intercostal insertion and retraction forces using ex vivo, food-grade, full-thickness porcine chest wall tissue from adult pigs at room temperature. Insertions and retractions were performed manually, perpendicular to the tissue surface, with the needle–catheter unit connected to a force measurement device (SAUTER FH 500, Kern & Sohn). Each measurement was repeated n=25 times at different insertion points. The mean of the peak insertion forces was 5.2*N* and had a standard deviation of 1.0.

It must be noted that the porcine tissue was thinner (20–34 mm) than typical human chest wall thickness, which for 95% of patients is below 64.4 mm at the second intercostal space, mid-clavicular line [19]. Using 20 mm as the worst case, the ratio between human anatomical thickness and sample thickness is 3.22. Assuming similar tissue properties, required peak forces can be approximated by scaling the measured values with this factor. From this scaling, the required peak insertion force for human application was derived as 26.4*N*. In addition, the manual insertion method introduces variability because insertion force depends on velocity. While higher speeds demand greater force, this effect plateaus at high velocities, and robotic insertions at constant speed have been reported to produce higher forces than manual insertions [20]. Based on these considerations, an insertion speed of approximately  is defined for the proposed robotic setup, ensuring that insertion is completed within about one second to minimize risks from patient movement during the procedure. The required insertion force is therefore approximated as 30 N.

### Mechanical design

Figure 1a presents the modular architecture of the end effectors subsystems. The end effector consists of an insertion cartridge, a quick-switch flange, a disinfectant cartridge, and a main fixture to which the ultrasound probe is attached. The main fixture comprises additionally an actuation motor and a tool-side quick-switch interface. The subsystems are described briefly in the following.

#### Insertion cartridge

**Fig. 3:**
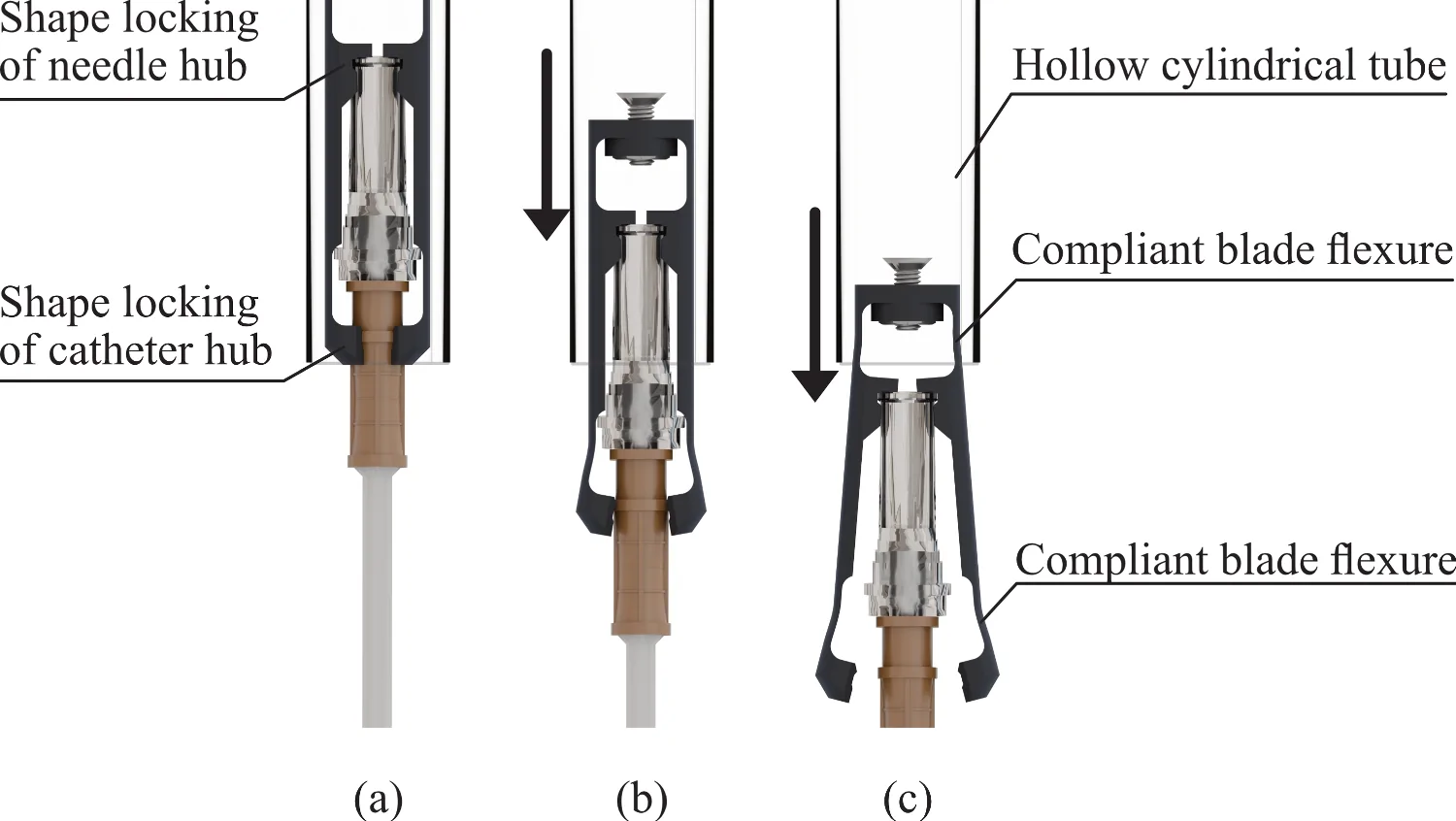
Gripper handling of a needle–catheter unit. (**a**) The gripper is fully inside the guide tube. (**b**) The gripper is partially protruding from the guide tube. The bottom compliant blade flexure opens the lower part of the jaw due to elastic relaxation and releases the catheter hub without releasing the needle hub. (**c**) The gripper protrudes almost entirely from the guide tube. The top and bottom flexures relax and open the jaws entirely. The needle hub and catheter are both released in this position

The main element of the insertion cartridge is the gripper used for gripping as well as insertion and retraction of the needle–catheter unit. The gripper was manufactured using fused deposition modeling (PLA). The flexible gripper employs a shape-locking principle to ensure secure fixation of the needle hub. Shape locking is realized through an end-stop that prevents retraction and a lower ridge that restricts forward motion. Jaw actuation is achieved via compliant blade flexures, which provide passive restoring force for opening, while closing relies on a high mechanical advantage generated by forcing the jaws into a hollow cylindrical tube (guide tube). In this configuration, the gripper remains normally open and attains reliable clamping when engaged at the bottom of the guide tube. Figure 3 shows the gripper design for handling of the needle–catheter unit.

**Fig. 4:**
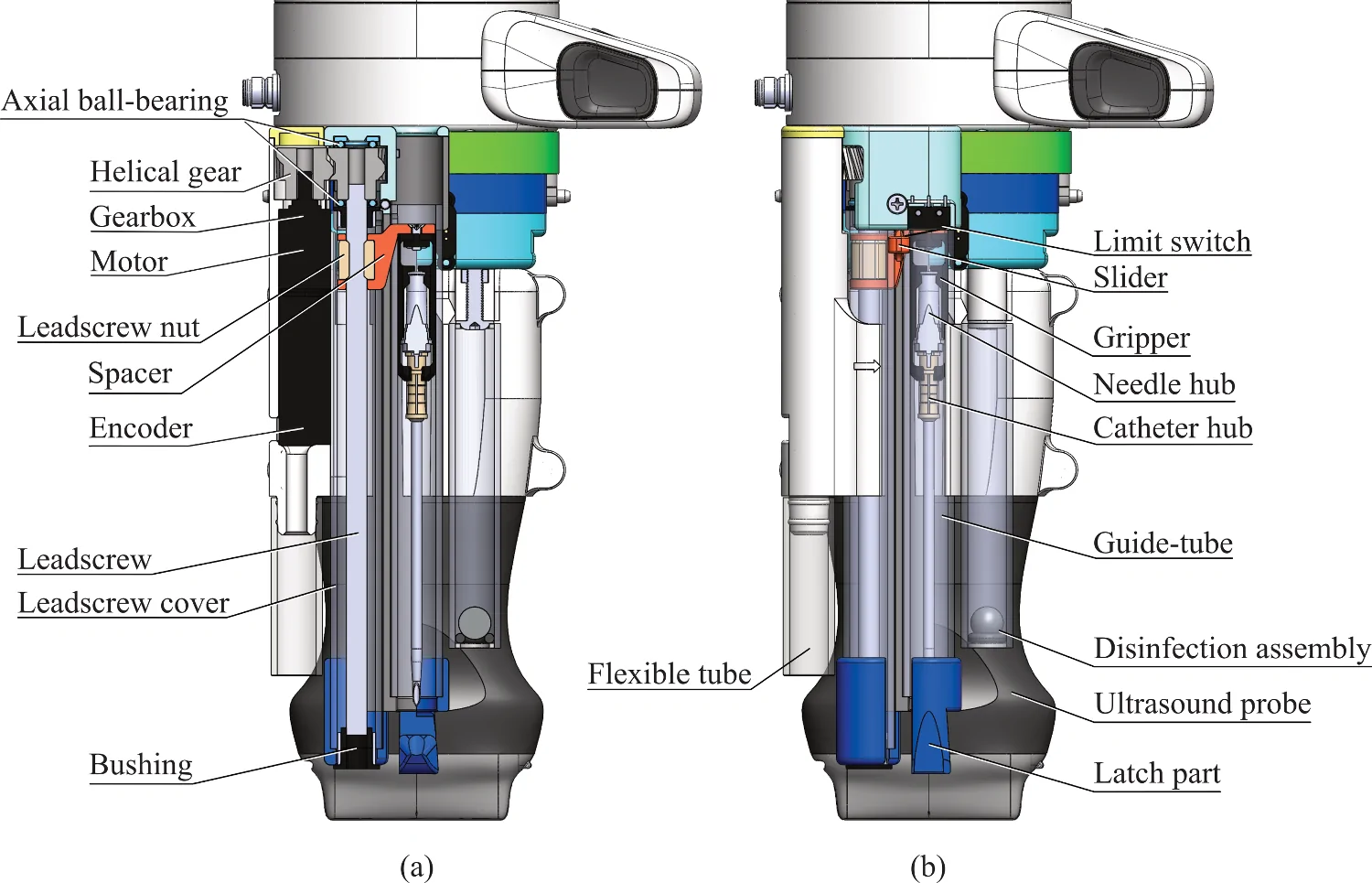
Actuator schematic of the end effector with frontal view (right) and frontal section through the needle insertion axis (left) of the developed end effector on the robot arm flange

The gripper is actuated by a motor in the main fixture, which drives a leadscrew in the insertion cartridge. The motor shaft is coupled to the leadscrew via a helical gear connection. The leadscrew drives a leadscrew nut that is constrained in rotation by a spacer component. The spacer transmits the motion of the leadscrew nut to the gripper. Figure 4 presents the actuator layout of the end effector.

**Fig. 5:**
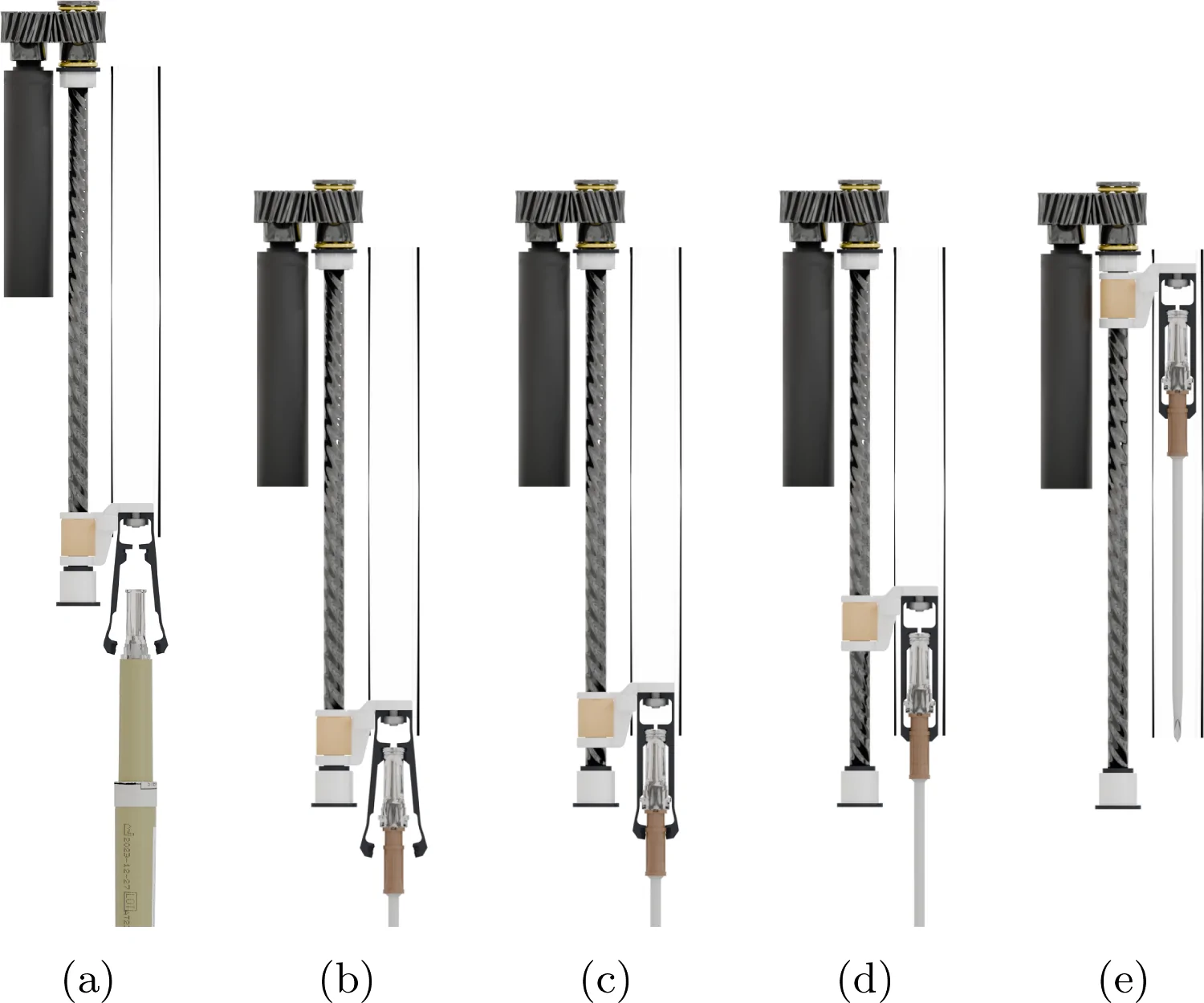
Phases (**a**–**e**) during pickup procedure for the developed gripper concept. The needle hub can be gripped directly from the sterile packaging tube

Further the presented gripper design allows pickup of the needle–catheter unit from sterile packaging. The pickup sequence and corresponding actuator and gripper motions are illustrated in Fig. 5. As only the needle hub initially protrudes, the flexible gripper jaws first engage the needle hub. Once lifted out of the sterile packaging tube, the catheter hub is also gripped analogously. For safety, the needle is fully enclosed within the guide tube at the end of the pickup procedure.

**Fig. 6:**
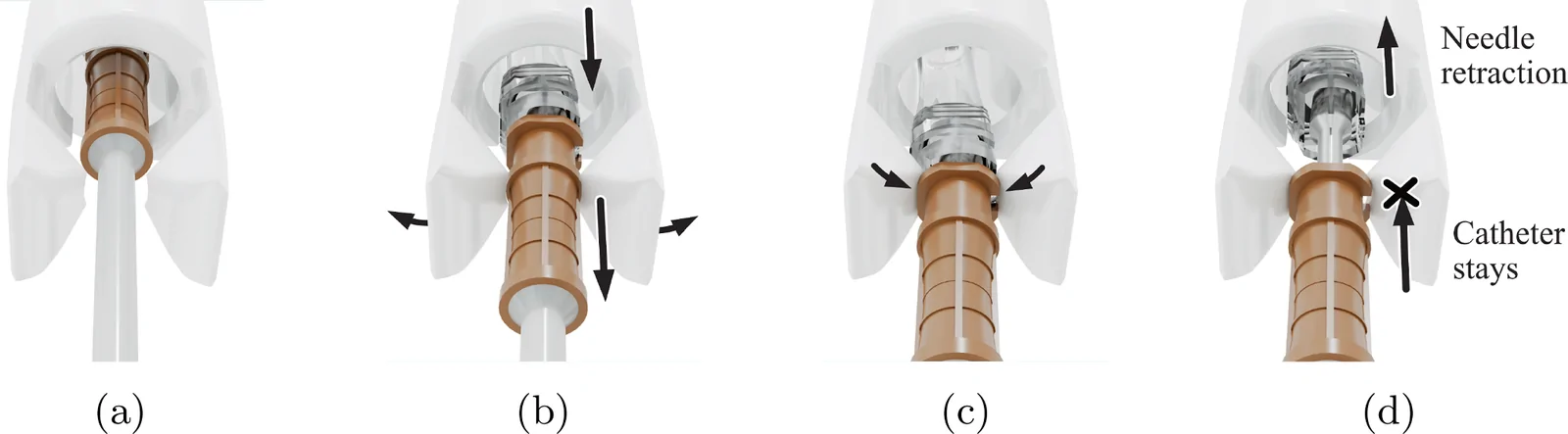
Passive blocking of catheter retraction during needle retraction using compliant latches

For needle insertion, the actuator advances the gripped needle–catheter unit along the guide tube toward the patient’s thoracic wall. The guide tube stabilizes the catheter and maintains alignment with the planned insertion axis, and prevents buckling of the gripper. As the needle inserts the intercostal space, the catheter follows passively until the pleural cavity is reached. During needle retraction, the catheter has to remain in situ. To allow independent motion between the needle and the catheter described in Sect. Task decomposition, a passive latch-based component is integrated. The component was manufactured using fused deposition modeling (PLA). This latch component is located at the bottom of the guide tube (see Fig. 4, right). During insertion, the advancing catheter overcomes the compliant latch force as shown in Fig. 6b and the catheter is inserted into the patient. During retraction, the latch blocks the catheter withdrawal, while allowing the needle to retract as shown in Fig. 6d.

#### Ultrasound probe

The ultrasound probe is mounted near the needle insertion axis, as shown in Fig. 4. It is used to identify the correct thoracic side by confirming pneumothorax presence and locate the upper rib edge within the intercostal space. The linear probe scans perpendicular to the ribs and parallel to the insertion cartridge, allowing rib landmarks to be identified by cranio-caudal motion and used to determine the insertion point. To reduce anatomical alignment errors, the offset between the ultrasound probe and insertion site is minimized to 28 mm. The primary limiting factor for further reduction in the offset is the thickness of the used ultrasound probe.

#### Disinfectant cartridge

Instead of ultrasound gel, a skin disinfectant is used as the acoustic coupling medium, in accordance with DEGUM recommendations [21]. This approach provides simultaneous disinfection of the puncture site while avoiding potential needle obstruction by gel. The disinfectant cartridge uses a passive, motion-actuated magnetic valve composed of a magnet, bearing ball, O-ring, and tube, allowing controlled fluid release through a defined robot motion pattern.

#### Quick-switch interface

A passive quick-switch interface enables automated mechanical and electrical connection of the tPTX module to robotic arms. Spring-loaded index pins and standard ISO flange compatibility allow fast tool changes, e.g., for clothing removal and decompression as described in Sect. Robotic workflow.

### Technical validation

To assess the feasibility of the proposed approach, the following technical validation focused on the mechanical performance of the needle–catheter unit and its interaction with the end effector. Full evaluation of the entire robotic workflow proposed in Sect. Robotic workflow was not pursued, as this would require integration of multiple complex subsystems, including teleoperation, imaging, and robotic path planning. Instead, the experiments targeted the most clinically relevant mechanical aspects of the decompression procedure, establishing a foundation for future system-level testing.

#### Critical load and load-dependent friction

The aim of the critical load experiment was to determine the permissible axial load on the gripped needle–catheter unit and to verify whether the developed gripper concept can withstand the required thoracic insertion forces. The measured load was compared with the estimated required insertion force in Sect. Insertion force and velocity constraints. Ten gripper samples were subjected to axial loading until failure. Each gripper was enclosed by a guide tube and engaged with a needle–catheter unit between its jaws. Axial displacement of the gripper was prevented, while a gradually increasing force was applied to the needle tip until structural failure occurred. The peak axial force $F_\mathrm{{Buckling}}$ at failure was recorded using a force measurement device. Frictional effects between the gripper and guide tube wall were neglected but analyzed in the following load-dependent friction tests.

The aim of the load-dependent friction experiment was to quantify the influence of axial preload on the frictional resistance between the gripper and the guide tube. Understanding this relationship is essential to evaluate potential force losses between the actuator and the needle tip, which directly affect insertion efficiency and control accuracy. Two preload conditions (30 N, and 100 N) were investigated to capture the behavior across a representative range of operational loads. For each preload setting, the peak force required to overcome static friction $F_\mathrm{{FrictionPeak}}$ was measured and compared to the applied preload force $F_\mathrm{{preload}}$. The relative wall friction force $F_\mathrm{{relWallFriction}}$ was calculated according to Eq. 1, providing a normalized measure of frictional effects as a function of axial load:

1 $$\begin{aligned} F_\mathrm{{relWallFriction}} = \frac{F_\mathrm{{FrictionPeak}}}{F_\mathrm{{preload}}} - 1. \end{aligned}$$

#### Needle-handling experiment

**Fig. 7:**
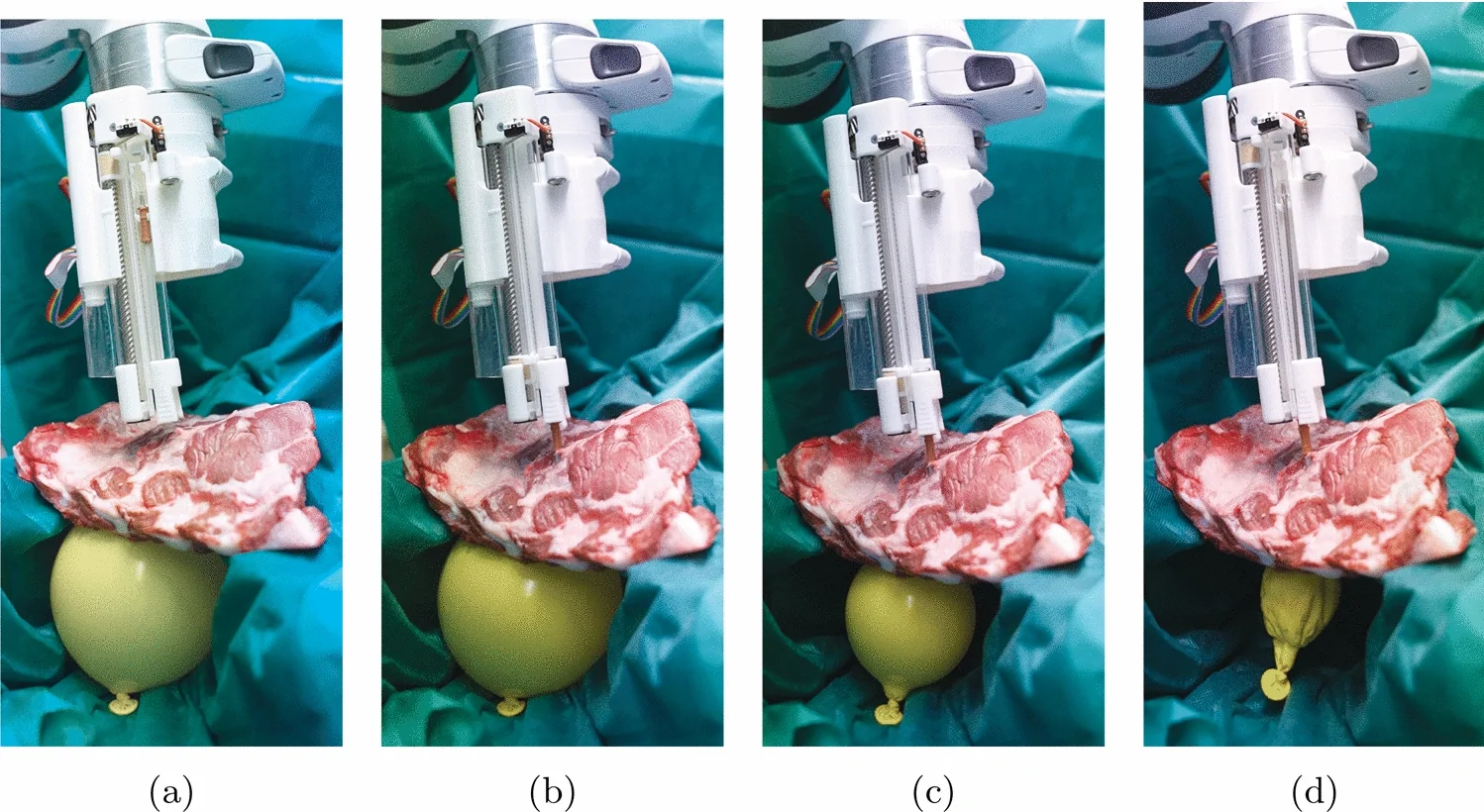
Example for successful robotic needle decompression procedure. (**a**) The tool is aligned with the sample such that the insertion axis coincides with an intercostal space. (**b**) The needle–catheter unit is inserted quickly into the sample. (**c**) During a 5–10s wait, the balloon begins to deflate. (**d**) The needle is retracted back into the tool and the catheter stays inside the tissue, while the balloon continues to depressurize

To evaluate the performance of the developed needle–catheter-handling approach under realistic conditions, an experiment was conducted using a ex vivo, food-grade, full-thickness porcine sparerib from adult pigs at room temperature. A balloon placed beneath the sparerib simulated pressurized air within the pleural cavity, enabling assessment of insertion, retraction, and sufficient insertion force. Therefore, the end effector was mounted to a robot, which remained in a fixed position, while a V-shaped platform allowed positioning of the sparerib model and balloon underneath, as illustrated in Fig. 7. Ultrasound and disinfectant modules were not used in this experiment. After insertion of a balloon in the platform, the sparerib was palpation-based aligned with an intercostal space under the needle–catheter unit. As described in the TCCC guidelines, the needle was inserted at approximately , hold for 5–10s, and retracted afterward. A successful trial required a full insertion of the needle–catheter unit overcoming the expected insertion force, a balloon puncture confirming decompression, and a full needle retraction while maintaining catheter position. Attempts striking ribs were marked invalid. The trials were repeated until n=10 successful insertions were achieved, or a maximum of $n_\mathrm{{max}}=20$ attempts was reached.

## Results

### Critical load and load-dependent friction

**Fig. 8:**
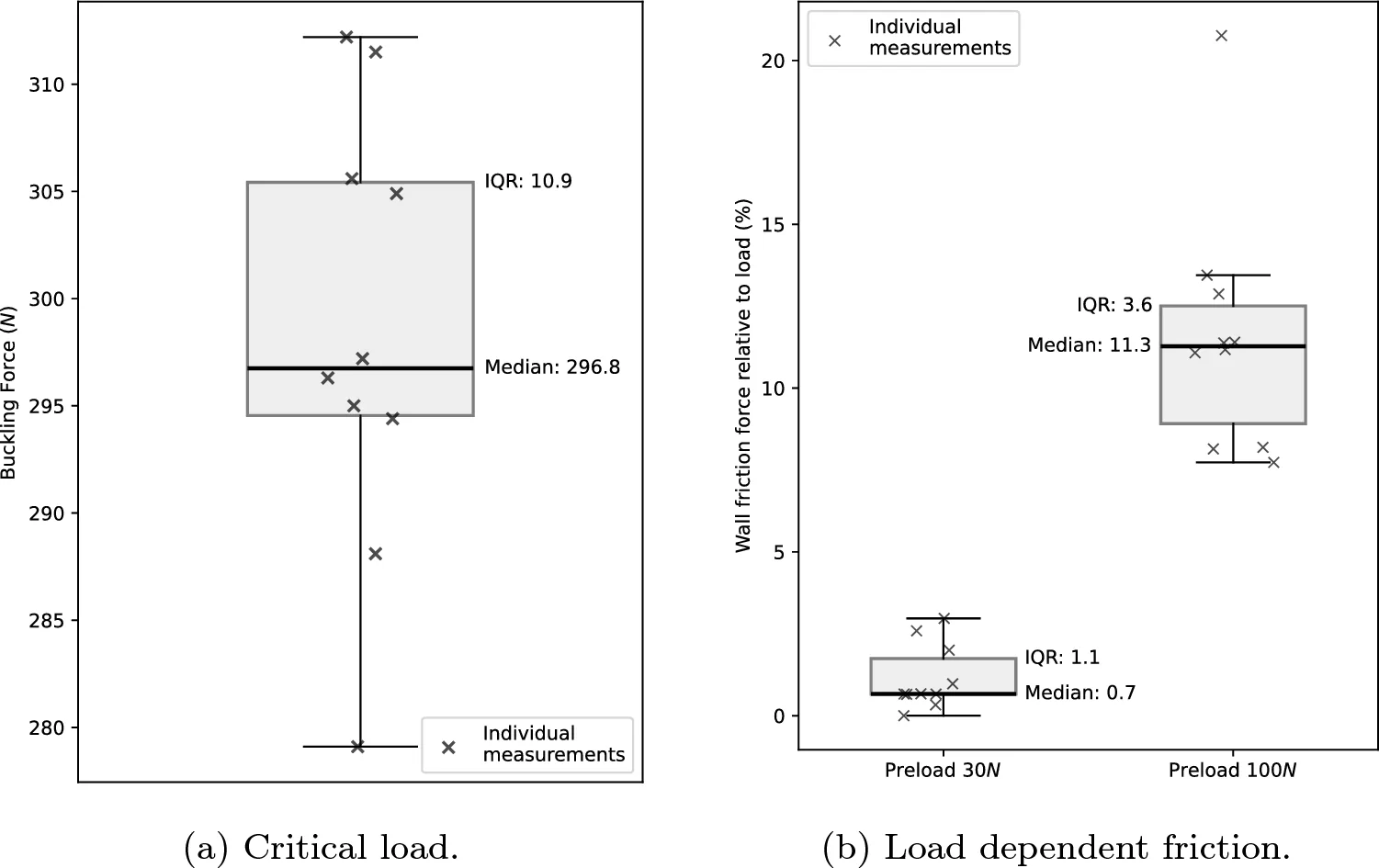
Results of the critical load and load-dependent friction experiments. (**a**) Results of the gripper force experiment. Box plot of the measured buckling force for the sample size of n=10. (**b**) Results of load-dependent friction experiment with sample size n=10 for the preload conditions 30 N and 100 N

Structural failure in the form of buckling was consistently observed across all n=10 gripper samples once the critical load was exceeded. The measured peak forces at failure are illustrated in Fig. 8a, with a mean of $F_\mathrm{{Buckling}} = 298.43 \pm 9.34$ N. Thereby, two distinct failure modes were identified: simultaneous buckling of both flexures near the needle hub, observed in one sample, and one-sided buckling, which occurred in the remaining nine samples. However, the results show that the gripper can safely withstand loads well beyond the required 30 N range without risk of buckling failure. Therefore, the developed gripper design provides a sufficient safety margin for thoracic needle–catheter insertion.

The results of the load-dependent friction experiment are presented in Fig. 8b. Each preload condition was tested with n=10 samples. The resulting wall friction force is expressed as a percentage of the applied preload. For a preload of 30 N, was $F_\mathrm{{relWallFriction}} = 1.15 \pm 0.95\%$. At a preload condition of 100 N, the mean relative friction increased to $F_\mathrm{{relWallFriction}} = 11.62 \pm 3.58\%$. For the preload condition of 0 N, the friction peak force was below the measurement threshold of the force measurement device. These results indicate that frictional losses increase with higher axial loading but remain within acceptable limits for system operation.

### Needle-handling experiment

To evaluate the functional performance of the developed end effector in a realistic scenario, a needle-handling experiment was conducted using an ex vivo porcine sparerib model to simulate the conditions of a clinical decompression procedure. Out of 11 valid trials, 10 met all success criteria, including full insertion of the needle–catheter unit, successful puncture of the balloon, and correct needle retraction with the catheter remaining in place, corresponding to a success rate of 90.9%. One attempt failed due to simultaneous retraction of the catheter and the needle after insertion. Seven additional attempts were excluded as invalid because the needle contacted a rib, likely resulting from inaccuracies in palpation-based alignment of the intercostal space. Post-experimental inspection of the failed sample revealed mechanical wear on one side of the compliant latch component, characterized by shearing between printed layers at the pinch point. The component was manufactured using fused deposition modeling (PLA), suggesting that the failure was influenced by the material’s limited mechanical strength. Overall, the results demonstrate reliable and repeatable robotic needle-handling performance under realistic experimental conditions.

## Discussion

Compared to the state-of-the-art, the presented needle–catheter-handling mechanism introduces a simplified yet effective approach to robotic tPTX decompression. By using a single linear actuator to generate two distinct relative motion patterns for the needle and catheter, the design achieves correct procedural sequencing with minimal mechanical complexity. This actuator-sharing concept eliminates the need for multiple perpendicular grippers as seen in earlier systems [10–12], thereby reducing overall size. Additionally, contact contamination is further minimized by limiting gripper contact exclusively to the sterile needle and catheter hubs. The experimentally determined intercostal insertion forces confirmed that the 10-gauge needle requires substantially higher insertion forces than previously reported for thinner 18-gauge models [9]. The measured mean insertion force supports a conservative design requirement of approximately 30 N for thoracic insertion, ensuring sufficient force capability for 95 % of the anatomical range. Correspondingly, the critical load experiments verified that the gripper structure can safely withstand loads far exceeding this value, demonstrating an adequate safety margin. Friction measurements revealed negligible losses (<2% at 30 N preload), confirming efficient force transmission through the guide tube. Collectively, these results validate the mechanical robustness of the proposed end effector design for performing needle decompression.

The needle-handling experiment demonstrated the feasibility of performing robotic intercostal insertions with high procedural accuracy. In 10 out of 11 valid trials (90.9%), the system achieved complete insertion, successful decompression, and correct retraction while maintaining the catheter in situ. The single failed trial resulted from partial latch wear, emphasizing the need for more durable materials and improved mechanical locking. Furthermore, the high number of invalid attempts due to rib contact highlights the importance of integrating ultrasound-based guidance for fine localization of intercostal spaces.

Several system components and functions were implemented conceptually but not yet tested in full robotic operation. This includes the automated opening of sterile packaging and pickup of the needle–catheter unit, which were only validated in preliminary manual trials. Similarly, the passive disinfectant dispensing valve, designed to serve for ultrasound coupling, requires further testing under robotic motion. Subsequent work should focus on validating these components under integrated robotic control to assess reliability, sterility, and workflow efficiency.

Future research will address the integration of imaging and control modalities necessary for fully teleoperated or semi-autonomous decompression. This includes ultrasound-based localization of insertion sites, robotic path planning with tissue-adaptive force control, and user interface design for intuitive remote operation. The addition of fast-reacting sensors for detecting airflow or temperature changes at the catheter tip could provide automated feedback confirming pleural decompression.

## Conclusion

Overall, this work establishes a foundation for telerobotic emergency intervention in tension pneumothorax. The proposed end effector demonstrates that reliable needle decompression can be achieved with minimal actuation and robust mechanical safety. With future integration of teleoperation, imaging, and automated verification, such systems could enable time-critical, life-saving intervention during transport of trauma patients to the hospital without onboard trained medical personnel.
